# Timing and technique impact the effectiveness of road‐based, mobile acoustic surveys of bats

**DOI:** 10.1002/ece3.3808

**Published:** 2018-02-18

**Authors:** Laura E. D'Acunto, Benjamin P. Pauli, Mikko Moy, Kiara Johnson, Jasmine Abu‐Omar, Patrick A. Zollner

**Affiliations:** ^1^ Department of Forestry and Natural Resources Purdue University West Lafayette IN USA; ^2^ Department of Biology Saint Mary's University of Minnesota Winona MN USA

**Keywords:** acoustic surveys, bat monitoring, bats, *Chiroptera*

## Abstract

Mobile acoustic surveys are a common method of surveying bat communities. However, there is a paucity of empirical studies exploring different methods for conducting mobile road surveys of bats. During 2013, we conducted acoustic mobile surveys on three routes in north‐central Indiana, U.S.A., using (1) a standard road survey, (2) a road survey where the vehicle stopped for 1 min at every half mile of the survey route (called a “start‐stop method”), and (3) a road survey with an individual using a bicycle. Linear mixed models with multiple comparison procedures revealed that when all bat passes were analyzed, using a bike to conduct mobile surveys detected significantly more bat passes per unit time compared to other methods. However, incorporating genus‐level comparisons revealed no advantage to using a bike over vehicle‐based methods. We also found that survey method had a significant effect when analyses were limited to those bat passes that could be identified to genus, with the start–stop method generally detecting more identifiable passes than the standard protocol or bike survey. Additionally, we found that significantly more identifiable bat passes (particularly those of the *Eptesicus* and *Lasiurus* genera) were detected in surveys conducted immediately following sunset. As governing agencies, particularly in North America, implement vehicle‐based bat monitoring programs, it is important for researchers to understand how variations on protocols influence the inference that can be gained from different monitoring schemes.

## 1 INTRODUCTION

Many bat species across the planet have experienced pronounced population declines (Mickleburgh, Hutson, & Racey, 2002; Ingersoll, Sewall, & Amelon, 2013). Habitat fragmentation and loss (Frey‐Ehrenbold, Bontadina, Arlettaz, & Obrist, 2013), the expansion of high‐intensity agricultural systems (Park, 2015), the proliferation of wind energy technology (Arnett & Baerwald, 2013), and disease (Thogmartin et al., 2013) are contributing factors to the diminishing numbers of bats worldwide. These threats necessitate methods to reliably survey bats in the environment, and acoustic monitoring has become an increasingly standard survey approach to address this need (Britzke, Gillam, & Murray, 2013). Growing popularity in acoustic surveying in the United States has been made possible through a combination of technological advances (Russo & Voigt, 2016) and the development of standardized protocols for collecting acoustic data on bats. For example, the U.S. Fish and Wildlife Service deemed acoustic monitoring to be an adequate method for determining presence of the endangered Indiana bat (*Myotis sodalis*) in surveys (Niver, King, Armstrong, & Ford, 2014). Outside of North America, acoustic monitoring of bats has been used to address research needs in Europe (Catto, 2004; Bellamy, Scott, & Altringham, 2013), Australia (Threlfall, Law, & Banks, 2012), Africa (Monadjem, Shapiro, Mtsetfwa, Reside, & McCleery, 2017), Asia (Hughes et al., 2010; Wordley, Foui, Mudappa, Sankaran, & Altringham, 2014), South America (Heer, Helbig‐Bonitz, Fernandes, Mello, & Kalko, 2015), and Central America (Jung & Kalko, 2010).

There are several techniques available to researchers using acoustic monitoring to survey bat activity or presence. Methods may be stationary where echolocation detectors are placed on the landscape at a fixed point and left to record for a specified amount of time or mobile where the researcher moves the detector by some means (e.g., walking, driving, and boating) along a predetermined route to record bat activity (Roche et al., 2011; Stahlschmidt & Brühl, 2012; Whitby, Carter, Britzke, & Bergeson, 2014). Active acoustic sampling relies on the researcher being present to point the detector microphone toward the area of greatest bat activity (Britzke, 2004), while passive sampling involves orienting the detector in the same direction for the entire sampling duration.

Britzke and Herzog (2009) outlined the first standard protocol for mobile, passive acoustic surveys to detect bats using vehicles in North America. During typical mobile acoustic surveys, researchers drive a sampling transect on low‐traffic roads at low speed. The detector is kept on throughout the entire transect, and a microphone is attached to the roof of the vehicle. During the sampling period, echolocation passes are recorded and their positions are georeferenced via GPS. This is the protocol used most widely among bat biologists implementing mobile surveys in North America (e.g., Loeb et al., 2015; Fisher‐Phelps, Schwilk, & Kingston, 2017) and is often used to generate indices of abundance for bat species (Loeb et al., 2015). However, other studies have used walking mobile acoustic surveys to investigate bat habitat selection (Ciechanowski, 2015), species presence (Bellamy et al., 2013), and diversity (Berthinussen & Altringham, 2012). Such measures could reasonably be ascertained from vehicle‐based mobile surveys as well (e.g., Whitby et al., 2014).

Vehicle‐based mobile transects offer several benefits as well as some costs relative to other sampling techniques. Mobile transects can sample a larger area in less time than a stationary method (Fisher‐Phelps et al., 2017), but such mobile sampling may require more effort on the part of investigators per unit observation than stationary acoustic sampling (Tonos, Pauli, Zollner, & Haulton, 2014). If the objective of a study is to develop a population abundance index, another advantage of vehicle‐based mobile surveys is that they allow for independent sampling of individuals as the detector is moving through space (Loeb et al., 2015). However, because sampling for driven mobile survey occurs only on roads or trails passable by motor vehicles, the data collected are inherently biased (Wellicome, Kardynal, Franken, & Gillies, 2014). It is known that some bats actively avoid roads with consistent vehicular traffic (Zurcher, Sparks, & Bennett, 2010; Bennett & Zurcher, 2013; Fensome & Mathews, 2016), and thus, a cost is that these species may be missed when conducting mobile surveys. Additionally, vehicles may interfere with the recording of bats due to the ultrasonic noise produced by cars (Schaub, Ostwald, & Siemers, 2008; Siemers & Schaub, 2011) or directly affect individual bats by deterring foraging due to vehicle‐produced noise or light (Bonsen, Law, & Ramp, 2015; Fensome & Mathews, 2016).

The timing of an acoustic survey of bats may have an impact on the effectiveness of that survey. Many bat species are known to have periods of increased activity during the night alternating with periods of relative inactivity (Catto, Racey, & Stephenson, 1995; Adams, McGuire, Hooton, & Fenton, 2015). To coincide with the peak activity of many bat species, most acoustic surveys begin at or near sunset (e.g., Britzke & Herzog, 2009). However, the difference in start time of just 1 hr (e.g., 30‐min presunset vs. 30‐min postsunset) has been shown to significantly affect the quantity of bat activity recorded (Goodenough, Deans, Whiteley, & Pickering, 2015). Thus, the timing of acoustic surveys may have important implications for the effectiveness of those surveys.

We hypothesized that modifying the standard mobile survey protocol used in North America in ways that reduce vehicular disturbance would improve the survey effectiveness. Specifically, we predicted that modifications would enhance detections of bats and improve numbers of echolocation passes recorded with enough quality for call identification to the genus level. We chose these measures as indices of survey effectiveness as any method that increases the number of identifiable calls would provide researchers with more power to detect changes in population trends or diversity. Our objectives were to implement two variations on the standard passive, mobile acoustic survey protocol: one where the vehicle stopped on the road to record echolocation passes for a time and one where a bicycle implemented the survey instead of a vehicle in an effort to reduce disturbance to bats associated with a vehicle (Zurcher et al., 2010). In addition, we hypothesized that the timing of the acoustic surveys, in accordance with previous research, would have an effect on the number of bat passes recorded. We predicted that the surveys conducted shortly after sunset would record more bat passes than those later in the night but that the difference between survey effectiveness would be rather moderate. Through these variations, we aimed to determine whether the number of identifiable bat passes per unit time spent surveying could be improved compared to use of the standard protocol.

## 2 MATERIALS AND METHODS

### 2.1 Study area

**Figure 1:**
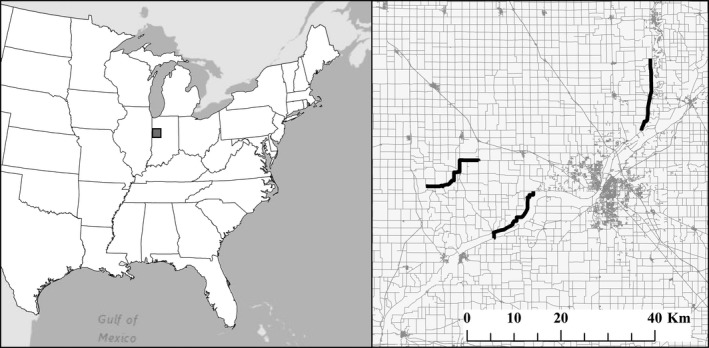
Location of the three routes sampled during 2013 in Indiana, USA, using variations on the standard mobile acoustic bat survey protocol. The left panel shows the eastern United States with the study area outlined by the gray box. The right panel displays the survey routes (black lines) and the roads (gray lines) within the study area

Our study area was located in north‐central Indiana (U.S.A.) across Carroll, Tippecanoe, White, and Warren counties. We selected three routes to conduct mobile transects in this area each ranging from 16.1 to 16.3 km in length (Figure 1). All routes were 2‐lane county roads with minimal traffic; the majority of the routes were paved, but some sections of routes were unpaved. The routes passed through a variety of different habitats including agricultural fields (corn or soybean), residential areas, forested areas, and stream corridors.

### 2.2 Acoustic surveys

We designed two new variations in sampling methods to test against the standard passive, vehicle‐based mobile acoustic protocol (deemed the “standard” method): The first, we named the “start‐stop” method, and the second was named the “bike” method. The standard protocol was modeled after Britzke and Herzog (2009) where an ultrasonic microphone was attached to the roof of a vehicle and a researcher drove the predefined route at speeds <40 km per hr. The start–stop method used the same method as the standard protocol except that at every 0.80 km (0.5 mi) along the route, the driver stopped on the road, turned off the engine and lights, and kept recording for 1 min. After the 1 min, the vehicle was started again and continued the route until the next stopping point. The bike method followed the standard protocol except in place of a vehicle, and a technician riding a bike with the detector placed in a front basket was used. A headlight was used with the bike method and traveling speeds matched the vehicle as much as possible as measured by use of a speedometer by the technician riding the bike. All three methods recorded continuously and traveled at <40 km/hr. We chose not to implement walking transects, common in Europe (Barlow et al., 2015), as our goal was to provide insights on modifications to the protocols being implemented in North America (Loeb et al., 2015) where surveys maintain a greater rate of speed to, presumably, sample individual bats only once.

Our surveys were conducted between 7 July and 15 August 2013. The first survey of each night began approximately 20 min after sunset in favorable weather conditions for maximum bat activity (no rain, wind speeds under 24 kph, temperatures above 10°C; Hayes, 1997). Routes were never concluded later than 3 hr after sunset. On each sampling night, we randomly selected a route to be sampled and randomly selected whether the start–stop or standard method would be completed first (due to the limitation of only a single vehicle). The direction traveled during each route was alternated between each survey night at a particular site. Once the first survey was completed, the vehicle returned to the beginning of the route to complete the next method, creating a 15‐min gap between nightly surveys. The bike method was always conducted simultaneously with the standard method (but not the start–stop method), but started at the opposite end of the route from the vehicle, crossing each other at the midpoint of the route. In total, 56 surveys were conducted during 20 survey nights—19 standard surveys, 19 stop–start surveys, and 18 bike surveys. Difference in surveys conducted was due to equipment failure.

For all vehicular methods, we used Anabat SD2 bat detectors (Titley Electronics, Bellini, New South Wales, Australia) with a microphone positioned at the top of the vehicle (height of approximately 2 m) pointing 5–15 degrees off‐vertical to decrease the collection of wind and other nonbat noise. The Anabat device was connected to a Personal Digital Assistant (PDA) device (HP iPAQ 212, Hewlett‐Packard Co, Palo Alto, CA) and GPS (GlobalSat CompactFlash SiRF STAR III Global Positioning System) unit which georeferenced recorded echolocation passes in real time. Both the start–stop method and the standard method were completed using a 2008 Chevrolet Colorado (Detroit, Michigan, U.S.A.). The bike method also used Anabat SD2 bat detectors housed inside a waterproof box (at a height of approximately 1 m) with the microphone outside of the box and oriented in the same manner as the vehicle‐based surveys. For each bike survey, a Garmin GPSmap60c was also attached to the detector to record route information, and georeferencing was completed post hoc manually by a technician. Anabat detectors were calibrated prior the surveys with an ultrasonic sound emitter to ensure equal sensitivity of detectors (Larson & Hayes, 2000). All detectors recorded with a data division ratio of 8 and sensitivity values near 7.

We used Analook (version 3.3q, Corben, 2002) to visually analyze and identify the bat passes that were recorded. A noise filter was used to distinguish bat passes from those files that contained only background noise (see Appendix S1). Only bat passes with >5 pulses and evidence of only one individual in the pass were retained in the analysis, as these are the characteristics of search‐phase bat calls that can be confidently categorized to species (Britzke & Murray, 2000). We elected this conservative measure of filtering the data for identification to avoid incorrect identification of bats with more variable call structure (e.g., eastern red bats, *Lasiurus borealis*), and those utilizing frequency shifting in the presence of another bat (Gillam & Montero, 2015). Using reference calls of species known to be in the area, we classified passes based on criteria outlined in Britzke and Murray (2000), limiting classifications to genus only. This was carried out to further limit the misidentification of bat passes between species that are closely related. The number of identifiable files was then divided by the total sampling time of that survey. This resulted in measures of the number of identifiable pass files per minute (for all genera combined and each genus separately) for each survey.

### 2.3 Data analysis

We created linear mixed models in program R (Version 3..3.2; R Core Team 2016) with the “lmerTest” (Kuznetsova, Brockhoff, & Bojesen, 2016) and “multcomp” (Hothorn, Bretz, & Westfall, 2008) packages to compare rates of bat detections per minute of sampling for each method. For each model, we treated the sampling method and whether the sampling occurred early (as the first survey of the night) or late as fixed effects and the site of sampling as a random effect. Models were created for all bat detections pooled together, those passes that could be identified to genus pooled together and each genus separately identified. To identify pairwise differences between the survey methods and survey times, Tukey contrasts with single‐step adjusted *p*‐values were conducted on those data that showed significant (*p* < .05) effects in the mixed model.

## 3 RESULTS

Across 56 surveys (in 20 survey nights), we recorded 1,901 bat pass files, 1,051 of which were detected using the start–stop method, 580 using the standard method, and 270 using the bike method. We categorized 844 of the total bat passes as belonging to a specific genus. The majority of the nonidentified bat passes had to be discarded in the genus‐level analyses due to not meeting one of the standards for correct identification outlined within the methods. Of these, 459 bat passes were detected using the start–stop method, 209 using the standard method, and 176 using the bike method (Table 1).

**Table 1:** Descriptive statistics and mixed model results for number of calls per minute for each detected bat genus by method of sampling and time of survey

Analysis variables	Values (mean ± *SE*) by method					
	Standard (S)	Start–Stop (SS)	Bike (B)	*F*	*p* a	Multiple comparisons^a^
Overall (all passes)	0.582 (0.053)	0.713 (0.039)	1.15 (0.081)	9.317	**<.001**	**B > S, SS**
Overall (identifiable)	0.165 (0.012)	0.241 (0.014)	0.172 (0.011)	3.207	**.049**	SS > B, S
*Eptesicus spp*.	0.048 (0.007)	0.072 (0.007)	0.034 (0.005)	2.532	.090	
*Lasiurus spp*.	0.074 (0.006)	0.118 (0.008)	0.088 (0.006)	2.834	.068	
*Myotis spp*.	0.016 (0.002)	0.030 (0.004)	0.029 (0.004)	1.264	.291	
*Perimyotis spp*.	0.028 (0.004)	0.021 (0.003)	0.020 (0.002)	0.501	.609	

	Values (mean ± *SE*) by survey time				
	Early (E)	Late (L)	*F*	*p* a	Multiple comparisons^a^
Overall (all passes)	0.827 (0.063)	0.792 (0.072)	0.059	.810	
Overall (identifiable)	0.233 (0.011)	0.156 (0.013)	8.764	**.005**	**E > L**
*Eptesicus spp*.	0.069 (0.007)	0.035 (0.006)	7.488	**.009**	**E > L**
*Lasiurus spp*.	0.111 (0.006)	0.077 (0.008)	4.283	**.043**	**E > L**
*Myotis spp*.	0.031 (0.004)	0.019 (0.004)	2.262	.139	
*Perimyotis spp*.	0.021 (0.003)	0.025 (0.003)	0.297	.588	

a Bold text indicates significance at α ≤ 0.05.

**Figure 2:**
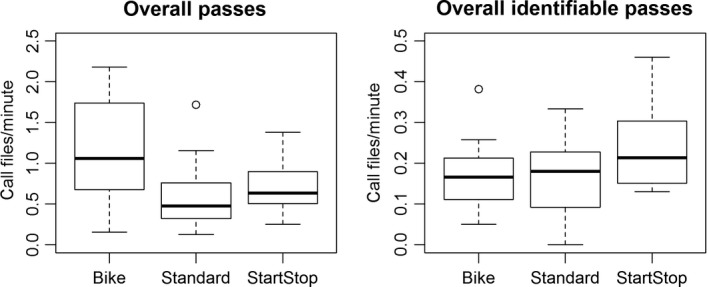
Box plots showing number of call files per minute for each method for all bat passes pooled together and for all identifiable (to the genus level) bat passes pooled together

The mixed model for all bat passes combined (regardless of identifiability) revealed a significant effect of sampling method on the number of passes detected per minute with the bike method recording more passes per unit time than both the standard method (*z* = 4.128, *p* < .001) and the start–stop method (*z* = 3.193, *p* = .004). The sampling method was also determined to have a significant effect on the number of identifiable bat passes recorded per minute. While the stop–start method recorded the greatest number of calls per minute, it was not significantly distinct from either the standard method (*z* = 2.288, *p* = .058) or bike method (*z* = 2.084, *p* = .093, Table 1, Figure 2). The method of sampling had no effect on the number of genus‐specific calls recorded per unit time though this effect neared significance for the *Myotis* and *Perimyotis* genera (Table 1).

**Figure 3:**
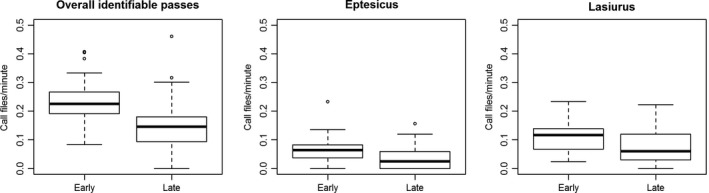
Box plots showing number of call files per minute detected in surveys conducted 20 min after sunset (early) or >1 hr after sunset (late) for all identifiable (to the genus level) bat passes pooled together and for the *Eptesicus* and *Lasiurus* genera individually

For three analyses, the timing of the survey was also a significant predictor of number of bat passes recorded: for overall identifiable bat passes, the *Eptesicus* genus‐level analysis, and the *Lasiurus* genus‐level analysis. Pairwise comparisons for all analyses revealed that a survey conducted early (within 20 min of sunset) detected significantly more bat passes than a survey conducted late (1 hr after sunset; Table 1, Figure 3).

## 4 DISCUSSION

It is important for researchers to understand how variations on a standard protocol will influence the data collected. Whitby et al. (2014) investigated differences between standard vehicle‐based mobile surveys and those conducted on a boat, concluding that boats did not offer advantages in data collection to the car‐based mobile surveys. Our study revealed using a bike to complete a mobile survey increased the number of bat passes recorded, but many of these additional recorded passes were of insufficient quality to be identified. However, when we implemented a start–stop protocol, we found a nearly significant difference where the number of identifiable bat passes detected per unit of sampling time increased.

We hypothesized that the lack of sound disturbance from a bicycle would increase detections of bats. We did detect more total bat passes with the bike method than either the start–stop or standard method, but these differences were not retained once genus identifications were completed. The bike method therefore recorded many fragmented and low‐quality passes not suitable for identification. One reason why the passes collected using a bike were of lower quality could be the difference in microphone height between the two methods. Research has shown that detector height can influence the amount of activity recorded (Collins & Jones, 2009) or which species groups are detected (Staton & Poulton, 2012). Further elevating detector microphones might increase call quality both to provide closer proximity to echolocating bats and to be farther from noise‐producing vehicles and echo‐producing impervious surfaces. The discrepancy in microphone height between the bike and standard method may have introduced variation in detection that obscured any differences due to the reduction in vehicle disturbance.

Our results indicated a general, but not statistically significant, increase in identifiable calls when the stop–start method was used. There are a number of reasons why the start–stop method may have been able to detect more identifiable bat passes than the standard method. The start–stop method may have detected more bat passes from the genera *Lasiurus* and *Eptesicus* because these are open‐habitat genera that often forage above the tree canopy (Menzel et al., 2005). Stopping the vehicle at intervals thus created a greater chance for individuals to move into the range of the recording microphone. By shutting off the vehicle's engine and lights, we may have reduced the disturbance effect of the vehicle. There is evidence that bats avoid crossing roads with moderate to heavy traffic, instead opting to travel along edge habitat to reach their foraging grounds (Russell, Butchkoski, Saidak, & McCracken, 2009). In one study investigating bat avoidance behavior along roads, researchers found that when vehicles were present, 60% of the bats observed avoided crossing the road versus 32% avoiding the road when no vehicles were present (Zurcher et al., 2010). The start–stop method may also be better at recording higher quality passes, thus improving our ability to identify recordings to the genus level. While stationary, the start–stop method would record bat echolocation without the excess noise from the vehicle, thus allowing clearer recordings of high‐frequency bats that attenuate more quickly (Wund, 2006). It is important to note, however, that while method significantly affected identifiable calls recorded, the stop–start method did not reach the level of statistical significance over either other method. Therefore, appropriate caution should be used when interpreting and extrapolating upon these results.

We found that for overall identifiable bat passes and for bat passes of *Eptesicus* and *Lasiurus*, the timing of the survey significantly influenced the number of passes recorded. The surveys that were started early (within 20 min of sunset) recorded more bat passes than those that were started late (>1 hr after sunset). Big brown bats (*Eptesicus fuscus*) are known to engage in only one foraging bout within the first few hours of sunset, with activity dropping off steadily as the night continues (Kunz, 1973). This reflects the importance of a survey recording during peak bat activity, typically defined as 30 min after sunset (Goodenough et al., 2015). Although not significantly different, other surveys also showed a higher mean number of bat passes recorded when the survey was completed first rather than second.

Our results can translate into several practical recommendations for ecologists wishing to monitor bats using acoustic monitoring, dependent on the study objectives. Conducting mobile surveys one after another in one night may cause researchers to miss the peak activity pulse of some bat species during the later survey, thus increasing the chance of missing bat passes that are present. While stopping along the transect may violate an assumption of independent detections of individual bats, similar analyses used to estimate an index of abundance for birds have been developed (Sauer, Link, Fallon, Pardieck, & Ziolkowski, 2013). The start–stop method emulates the North American Breeding Bird Survey (BBS), a roadside survey completed largely by volunteers that have tracked bird population trends since the 1960s (Sauer et al., 2013). Matching the BBS has advantages for bat researchers, as this method has been successfully implemented for several decades and statistical models using data collected in this way are well‐established (Sauer et al., 2013). Unlike with the BBS, volunteers collecting bat echolocation data need not be experts in identification to complete the surveys (Miller et al., 2012), thus this method may be even more valuable to bat researchers wishing to conduct large‐scale population monitoring, possibly using citizen scientists in the field.

Mobile surveys can be used to answer important research questions other than long‐term population monitoring. For presence/absence surveys in an occupancy modeling context, the start–stop method could combine the benefits of stationary and mobile methods. Stationary acoustic monitoring has been shown to detect rare species better than traditional mist‐netting surveys (O'Farrell & Gannon, 1999) or mobile surveys (Tonos et al., 2014; de Torrez, Wallrichs, Ober, & McCleery, 2017). Stopping along points in a transect could increase detection probabilities for rare species by improving the quality of recorded passes for identification. Additionally, implementing a start–stop mobile survey could improve measures of diversity and richness in census surveys across large areas (Murray, Britzke, Hadley, & Robbins, 1999). We suggest further investigations on how the start–stop method compares in detecting temporal changes of bat activity during long‐term monitoring programs.

Our study used only Anabat SD2 detectors to test modified methods against the standard mobile protocol. However, there are a wide variety of bat echolocation recorders available to researchers (Adams, Jantzen, Hamilton, & Fenton, 2012). Anabat SD2 detectors record using a zero‐crossing algorithm, but other models can record in full spectrum or heterodyne modes and such additional information may be particularly important in species‐rich communities where additional data on call structure are necessary for call identification. These additional modes of recording retain different information within the echolocation files and could potentially produce different results as they provide more information on call structure to the researcher which can aid in identification (Adams et al., 2012). Thus, extrapolation of our conclusions to other recording devices should be carried out with caution. We suggest a similar exploration of these methods for full spectrum and heterodyne recording devices. It is also important to note that this study used the number of calls recorded per minute of sampling as a measure of method effectiveness. This measure is intuitively valuable to researchers as it allows for the maximization of recorded bat passes during a set sampling period. However, other measures such as the coefficient of variation between samples or measures of species diversity may be more important in some research contexts (Whitby et al., 2014), and the effectiveness of the techniques presented in the manuscript may differ for those variables.

Large‐scale monitoring of bat populations has been a research need identified by bat biologists for over a decade (O'Shea, Bogan, & Ellison, 2003). In response to declining populations across the world, many government agencies have begun to design and implement monitoring programs that use mobile surveying. In Ireland, car‐based surveys are an integral part of the bat population monitoring program (Catto, 2004). In North America, a multiscale continent‐wide project, the North American Bat Monitoring Program (NABat), has been developed that uses stationary and mobile car surveys to track bat population trends (Loeb et al., 2015). As these monitoring programs become established, it is important that researchers participating in such programs have an understanding of the ways in which variations on vehicle‐based mobile survey methods can affect the data collected with the goal of deploying those methods that best meet research objectives.

## CONFLICT OF INTEREST

None declared.

## AUTHOR CONTRIBUTIONS

LD contributed to data collection, analysis, interpretation and was primary author of text. BP designed experiment and aided in data collection, analysis, interpretation and revisions of text. MM contributed to data collection, analysis, interpretation, and writing of text. KJ contributed to data collection, analysis, interpretation, and writing of text. JA contributed to data collection, analysis, interpretation, and writing of text. PZ oversaw project and contributed to experimental design, data analysis, interpretation, and revision of text.
