# Supplementary material for: Timing and technique impact the effectiveness of road‐based, mobile acoustic surveys of bats
Source: Ecol Evol. 2018 Feb 18;8(6):3152–60. doi: 10.1002/ece3.3808 (PMC5869262; doi:10.1002/ece3.3808)
Supplement: Supplementary file 1 [file ECE3-8-3152-s001.docx]

Appendix S1: Filter settings used in analysis of echolocation calls in Analook 3.3q.

| **Parameter** | **Value** |
| --- | --- |
| buzz | 0 |
| smooth | 15 |
| maxposchg | 9999 |
| maxnegchg | 9999 |
| highstart | 0 |
| lowstart | 0 |
| alldrop | 0 |
| minNtrans | 0 |
| mindur | 1000 |
| maxdur | 999000 |
| minFmax | 4000 |
| maxFmax | 300000 |
| minFmin | 4000 |
| maxFmin | 300000 |
| minFmean | 4000 |
| maxFmean | 300000 |
| bodyover | 240 |
| minFc | 4000 |
| maxFc | 300000 |
| minsweep | 6000 |
| maxsweep | 300000 |
| minS1 | -9999 |
| maxS1 | 9999 |
| minSc | -9999 |
| maxSc | 9999 |
| minNcalls | 5 |
| Tforcalls | 15000000 |
| maxstrtlin | 9999 |
| maxstrtdev | 9999 |
| minstrtdur | 0 |
| maxstrtdur | 999000 |
| minstrtslope | -9999 |
| maxstrtslope | 9999 |
| maxbuzzdur | 0 |
| maxbuzzsep | 0 |
| minbuzzN | 999 |
| minbuzzlen | 0 |
| maxbuzzlen | 999000 |
| minbuzzFmax | 4000 |
| minbuzzFmean | 4000 |
| maxbuzzFmean | 300000 |
